# Development of a generative deep learning model to improve epiretinal membrane detection in fundus photography

**DOI:** 10.1186/s12911-024-02431-4

**Published:** 2024-01-26

**Authors:** Joon Yul Choi, Ik Hee Ryu, Jin Kuk Kim, In Sik Lee, Tae Keun Yoo

**Affiliations:** 1 Department of Biomedical Engineering, Yonsei University, Wonju, South Korea; 2 Department of Refractive Surgery, B&VIIT Eye Center, B2 GT Tower, 1317-23 Seocho-Dong, Seocho-Gu, Seoul, South Korea; 3 Research and development department, VISUWORKS, Seoul, South Korea

**Keywords:** Epiretinal membrane, Fundus photography, Deep learning, Generative adversarial net

## Abstract

**Background:**

The epiretinal membrane (ERM) is a common retinal disorder characterized by abnormal fibrocellular tissue at the vitreomacular interface. Most patients with ERM are asymptomatic at early stages. Therefore, screening for ERM will become increasingly important. Despite the high prevalence of ERM, few deep learning studies have investigated ERM detection in the color fundus photography (CFP) domain. In this study, we built a generative model to enhance ERM detection performance in the CFP.

**Methods:**

This deep learning study retrospectively collected 302 ERM and 1,250 healthy CFP data points from a healthcare center. The generative model using StyleGAN2 was trained using single-center data. EfficientNetB0 with StyleGAN2-based augmentation was validated using independent internal single-center data and external datasets. We randomly assigned healthcare center data to the development (80%) and internal validation (20%) datasets. Data from two publicly accessible sources were used as external validation datasets.

**Results:**

StyleGAN2 facilitated realistic CFP synthesis with the characteristic cellophane reflex features of the ERM. The proposed method with StyleGAN2-based augmentation outperformed the typical transfer learning without a generative adversarial network. The proposed model achieved an area under the receiver operating characteristic (AUC) curve of 0.926 for internal validation. AUCs of 0.951 and 0.914 were obtained for the two external validation datasets. Compared with the deep learning model without augmentation, StyleGAN2-based augmentation improved the detection performance and contributed to the focus on the location of the ERM.

**Conclusions:**

We proposed an ERM detection model by synthesizing realistic CFP images with the pathological features of ERM through generative deep learning. We believe that our deep learning framework will help achieve a more accurate detection of ERM in a limited data setting.

**Supplementary Information:**

The online version contains supplementary material available at 10.1186/s12911-024-02431-4.

## Background

An epiretinal membrane (ERM), also known as an epimacular membrane or macular pucker, is an abnormal semi-translucent film of fibrocellular tissue at the vitreomacular interface (over the internal limiting membrane) [1]. Clinical presentations of ERM include: decreased visual acuity, metamorphopsia, micropsia, and monocular diplopia. However, most patients with ERM are asymptomatic at early stages. The prevalence of ERM generally increases with age. According to a previous report, 30 million adults in the United States have ERM. In a nationwide study in South Korea, the prevalence of ERM was reported as 2.9–7.0% [2, 3]. The prevalence rate is expected to increase in aging societies. ERM can be treated by vitreoretinal surgery using a pars plana vitrectomy procedure and membrane peeling [4]. If the fibrocellular tissue is detected early and removed by surgery before vision decreases, vision loss can be prevented. Most ERMs have no specific causes. Therefore, screening for ERM will become increasingly important.

**Fig. 1:**
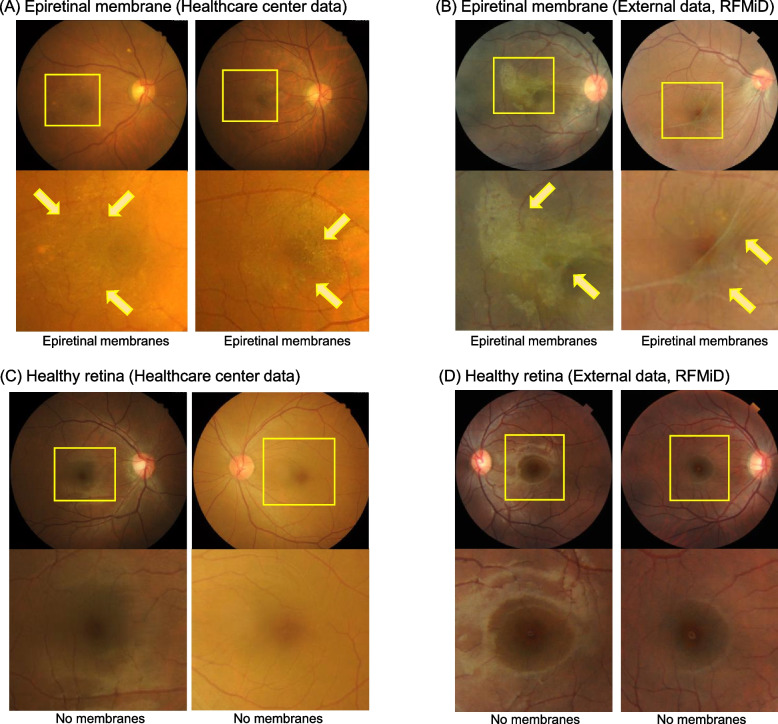
Representative fundus photographs (FPs) of the abnormal semi-translucent film of fibro-cellular tissues of epiretinal membranes (ERM) with reduced visual acuity and healthy retinas. **A** FP with ERM from the healthcare center data. **B** FP with ERM from the external validation data. **C** FP with healthy retina from the healthcare center data. **D** FP with healthy retina from the external validation data

Recently, the detection of ERM using optical coherence tomography (OCT) was established [1]. OCT reveals a hyperreflective layer of the fibrocellular membrane tissue by directly imaging the vitreoretinal interface. However, OCT is unsuitable as a retinal screening method because of its relatively long measurement time and difficulty in configuring the equipment. ERM can be diagnosed based on fundus examination or color fundus photography (CFP), as shown in Fig. 1. The cellophane reflex in the macular area can be observed by careful examination of eyes with ERM [5]. There can be an irregular foveal contour or a wrinkled retinal surface due to contracture of the fibrocellular membrane. However, because the membrane tissue is transparent, it is possible to misdiagnose ERM using fundus photographs. Most studies using artificial intelligence (AI) to diagnose ERM have concentrated in the OCT image domain [6, 7].

**Fig. 2:**
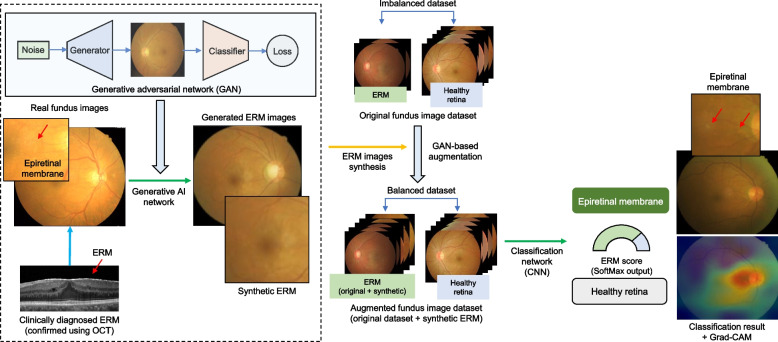
Schematic diagram of the development of deep learning model for epiretinal membrane (ERM) detection. The generative adversarial network (GAN) model augments ERM images with proper diversity and high quality to improve diagnostic performance. After augmenting the training data for ERM, we trained deep learning networks via transfer learning to classify ERM and healthy retinas

Considering the high prevalence of ERM, few AI-based studies have attempted to investigate ERM detection in the CFP domain compared to many other studies on diabetic retinopathy, age-related macular degeneration, and glaucoma [8, 9]. A previous study focused on the diagnosis of ERM through CFP using deep learning; however, the accuracy was relatively low [10]. This low accuracy was attributed to the relative lack of CFP data with ERM. Several previous studies on a big-data scale have analyzed ERM as a subclass for multiclass retinal disease classification [11–13]. Recently, generative artificial intelligence (AI) was introduced to overcome the lack of data on rare diseases [14]. In this study, we synthesized CFP images with ERM by using a generative AI technique (generative adversarial network; GAN). Using the augmented data generated by StyleGAN2, we improved the diagnostic accuracy of the deep learning models for detecting ERM (Fig. 2). To confirm the performance, we validate the models using external datasets.

## Methods

### Data collection

We retrospectively collected CFP data containing ERM from an Eye Care Center (B&VIIT Eye Center, Seoul, South Korea). This study was approved by the Institutional Review Board of the Korean National Institute for Bioethics Policy (KNIBP) and the requirement for informed consent was waived. All procedures were performed in accordance with the ethical standards of the institutional and national research committees and the 1964 Declaration of Helsinki and its later amendments or comparable ethical standards. The clinical data of human participants, except for CFP, were not obtained in this study. We collected CFP images from patients with ERM diagnosed with the KCD code H3539 (IDC-10 code H35.379) between January 2015 and December 2022. External validation was conducted using publicly accessible CFP databases to validate the developed deep learning models. The external databases include: the retinal fundus multi-disease image dataset (RFMiD) [15] and the Joint Shantou International Eye Center dataset (JSIEC) [12].

**Fig. 3:**
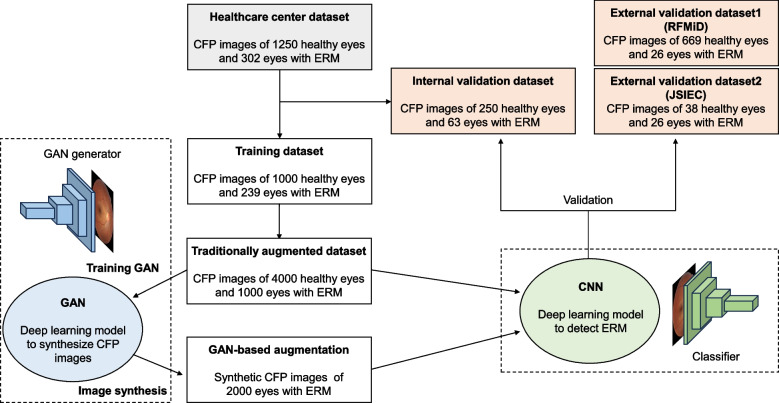
Dataset used in developing and validating the epiretinal membrane detection model in fundus photography. The deep learning models were trained and internally validated using randomly partitioned 80 and 20% of data, respectively. Using the training dataset, GAN models were trained to increase the volume of the ERM dataset for data augmentation. We finally built an ERM detection model based on the GAN augmentation techniques. The two external validation datasets, including RFMiD and JSIEC, represented a real scenario of a check-up center with CFP screening

Data processing is demonstrated in the Supplementary Materials. The healthcare center dataset consisted of CFP images of 1,250 healthy eyes and 302 eyes with ERM. The training and internal validation datasets were obtained using healthcare center data and were randomly split. We assigned 1,239 CFP images (80%, including 1000 healthy and 239 ERM) to the training dataset, and 313 images (20%, including 250 healthy and 63 ERM) were used as the internal validation dataset. The GAN-based method augments ER images with proper diversity and high quality to improve diagnostic performance. After augmenting the training data for ERM, we trained deep learning networks via transfer learning to classify ERM separately from healthy retinas. Two external validation procedures were performed. We collected the RFMiD test set (669 healthy retinas and 26 ERM) and JSIEC dataset (38 healthy retinas and 26 ERM). The labels on the datasets from the healthcare center and publicly accessible sources were confirmed by an ophthalmologist. The data flow is shown in Fig. 3. We confirmed that the training of the GAN and convolutional neural network (CNN) models were performed using only the training dataset and that there was no overlap in the training (for both the GAN and CNN) and validation datasets, as shown in Fig. 3.

### GAN image synthesis

With recent vigorous research on generative AI, GAN has been established as a standard method for generating medical images [16]. As the GAN model learns the image pixel data distribution for data synthesis, the training dataset requires a sufficient volume to train the generator without mode collapse or overfitting. We attempted to overcome this problem of overfitting sing traditional augmentation techniques with simple geometric transformations. Traditional data augmentation was performed using linear spatial transformation including: left and right flipping, width/height translation from -5% to + 5%, random rotation from -15° to 15°, zooming from 0 to 15%, and random brightness change from -10% to 10%. Initially, we prepared 4000 healthy and 1000 ERM CFP images to train the GAN. As shown in Fig. 3, we attempted to improve the performance of the deep-learning classifiers by creating an additional 2000 synthetic CFPs with ERM using the GAN algorithm. It aims to eliminate data imbalance and further generalize the model by supplementing more diverse and realistic synthetic data through the GAN.

In this study, we adopted the deep convolutional GAN (DCGAN), CycleGAN, and StyleGAN2, which are the most popular GAN techniques in the medical field [17]. The DCGAN is a basic form of GAN architecture based on the vanilla GAN that replaces the building block of the generator with fully convolutional layers [18]. DCGAN has been successfully used to synthesize CFP images of glaucoma [19]. CycleGAN is the most popular unpaired image-to-image translation GAN technique [14]. The basic concept of CycleGAN is cyclic consistency, in which the training algorithm matches the features of the image data distribution between two classes in an unpaired dataset. CycleGAN was used to generate denoised CFP images from images with artifacts [20]. Recently, StyleGAN2 has been well-adopted to synthesize high-resolution images [21, 22]. StyleGAN employs the concepts of similarity and aversion. StyleGAN demonstrated good performance in synthesizing high-resolution CFP images [23]. StyleGAN2, which is an advanced version of StyleGAN, has been successfully adopted in the medical field for knee radiography and colonoscopy image synthesis [17, 24]. The sources of the backbone codes of the GAN architectures are shown in the Supplementary Materials section and were modified to adapt to CFP synthesis. The size of the output images was set to a resolution of 256 × 256 pixels to use the default architecture of the GAN models. The GAN models were trained using the same dataset. In our experience, owing to the limitations of the volume of data, the GAN model did not properly learn using only with CFP images of ERM. Therefore, the healthy retina data were learned together with the ERM to properly generate realistic CFP images. DCGAN and StyleGAN2 were trained by combining both healthy retinas and ERM data, and the generated ERM data were used for further deep-learning training. By contrast, CycleGAN separates healthy and ERM data to learn domain translation and generates ERM data by infusing pathological characteristics into healthy images. An ophthalmologist reviewed the CFP images generated by the GAN models and removed the synthetic images with artifacts or without ERM features. Only generated images that confirmed the structures of the optic disc and vascular arcades classical of ERM were used for training. This manual selection process was performed to improve the diagnostic performance of GAN-based augmentation. Finally, we generated 2,000 synthetic CFP images with ERM for each GAN technique to train the CNN models. The deep-learning models were trained using an NVIDIA RTX 2080Ti GPU with 4,352 CUDA cores and 11 GB of RAM.

### CNN model training

After the GAN-based augmentation to enrich the ERM data, we built a CNN classifier model for ERM detection. We used ResNet50 and EfficientNetB0 as the backbone CNN models for the classifiers. These architectures have been recognized as standard models owing to their robustness and performance [25]. The CNN architectures were pre-trained on general image features from the ImageNet data and imported into the workspace. The input images were resized to the input tensor of each original CNN architecture (224 pixels × 224 pixels for ResNet50 and EfficientNetB0). The last layers of the CNN architecture were replaced with a modified fully connected network layer (with 2 × 2048 weights and 2 × 1 bias) and two softmax functions for the two classes (ERM and healthy), which set the output of the prediction score to a range of zero to one, which corresponds to the prediction probability of each class. All CNN training procedures were optimized using stochastic gradient descent (SGD) with a momentum algorithm (SGD learning rate = 0.0001) and a mini-batch size of 20 over 100 epochs, which are the fine-tuning parameters for transfer learning. Using the Grad-CAM technique, attention heat maps were generated from the last layers of the softmax and the activation convolutional layers of the trained CNN model. This visualization indicates whether the CNN model was properly trained with a focus on the ERM features. To determine the best data-augmentation strategy, we trained the CNN weights using no augmentation, simple geometric transformation (classic augmentation) to balance the case–control datasets (ERM data oversampling), and GAN-based augmentation. For an additional comparison experiment, we adapted the denoising diffusion probabilistic model (DDPM) [26, 27] and CutMix [28] to augment the ERM data. A pretrained vision transformer (ViT) with transfer learning software [29] was used to check whether the performance could be improved.

### Statistical analysis

The performance of the CNN models for detecting ERM was evaluated using metrics including: the area under the curve (AUC) of the receiver operating characteristics (ROC), sensitivity, and specificity. Due to the characteristics of the imbalanced data, we adopted Youden’s index, a standard threshold method that assigns equal weights to sensitivity and specificity.

## Results

**Fig. 4:**
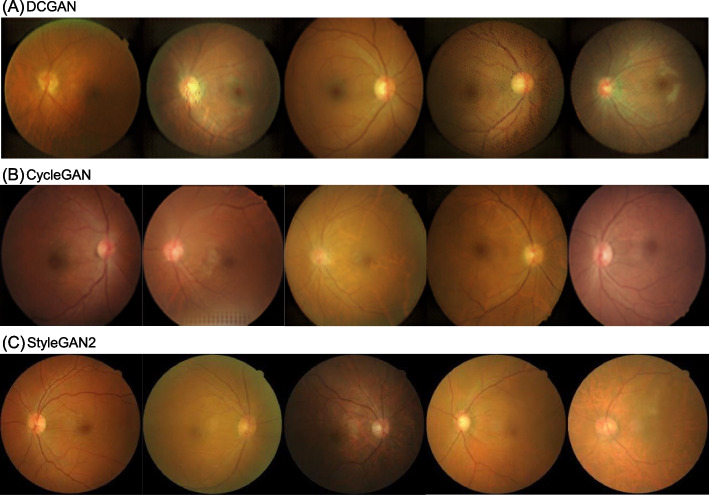
Epiretinal membrane image generation using generative AI algorithms. **A** DCGAN. **B** CycleGAN. **C** StyleGAN2

**Fig. 5:**
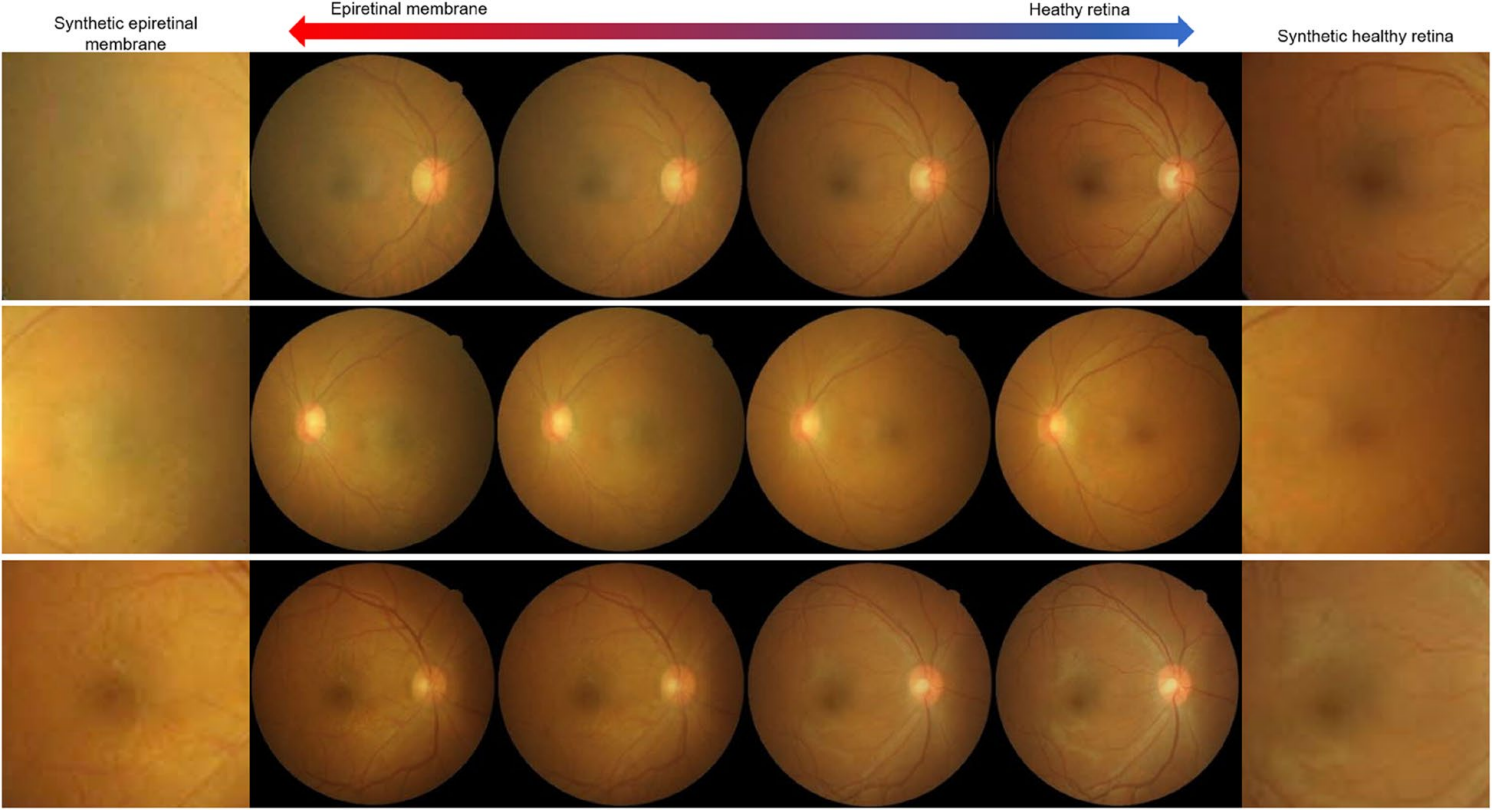
Synthetic fundus photographs according to latent space changes in the StyleGAN2 model

Initially, we trained the GAN models based on traditional augmentation. Figure 4 shows the representative results of GAN image generation. The CFP images with ERM synthesized showed the basic structures of the macula, with the optic nerve, vascular arcade, and fovea, for all GAN techniques. The synthetic images generated by the DCGAN were of low quality and had distinct artifacts. The synthetic images generated by the CycleGAN also had some checkerboard artifacts and showed insignificant ERM features. Compared to DCGAN and CycleGAN, StyleGAN2 synthesizes realistic CFP images with significant ERM features. After an ophthalmologist reviewed the images generated by the GAN models, we retained 2,000 CFP images with ERM for each GAN technique and added them to the original training dataset. As shown in Fig. 5, an ERM attribute can be infused into the CFP by adjusting the latent space of the trained StyleGAN2 in a certain direction. However, because ERM is not completely independent of other factors, other changes in CFP are associated with ERM generation.

**Fig. 6:**
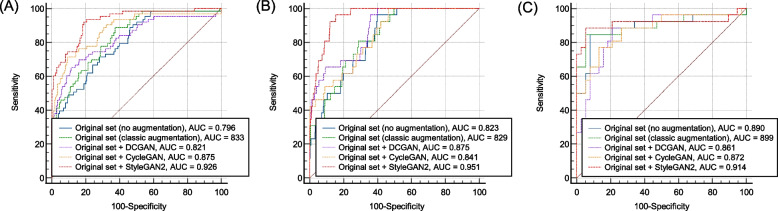
Validation results of ROC curves for detection of epiretinal membrane. **A** Healthcare center dataset. **B** External dataset 1 (RFMiD). **B** External dataset 2 (JSIEC)

Figure 6 shows the ROC curves for the ERM detection results of the EfficientNetB0 models for the internal and external validation results. Table 1 shows the ERM detection performance using the internal validation dataset. EfficientNetB0 trained with StyleGAN2 augmentation exhibited the best detection performance. The AUC of the proposed styleGAN2 method was 0.926 (95% confidence interval [CI], 0.890–0.963), which was better than that of the other models. It yielded a sensitivity of 92.0% (95% CI, 82.4–97.3%), a specificity of 80.8% (95% CI, 75.3–85.4%), a PPV of 54.7% (95% CI, 48.1–61.1), and an NPV of 97.5% (95% CI, 94.5–98.9%). In both ResNet50 and EfficientNetB0 architectures, augmentation with StyleGAN2 resulted in better AUCs than the other GAN techniques. The deep learning models with classic linear augmentation were inferior to EfficientNetB0 trained with StyleGAN2 augmentation.

**Table 1:** The prediction results from the internal validation (healthcare center dataset) to detect epiretinal membrane in fundus photographs

CNN architectures	Training set	ROC-AUC (95% CI)	Sensitivity (%, 95% CI)	Specificity (%, 95% CI)	PPV (%, 95% CI)	NPV (%, 95% CI)
ResNet50	Original set (no augmentation)	0.766 (0.702–0.830)	71.4 (58.6–82.1)	70.4 (64.3–75.9)	37.8 (32.2–43.7)	90.7 (86.7–93.6)
	Original set + classic augmentation^a^	0.827 (0.780–0.867)	92.1 (82.4–97.4)	58.0 (51.6–64.2)	35.6 (31.9–39.4)	96.7 (92.5–98.5)
	Original set + DCGAN	0.850 (0.798–0.902)	90.4 (80.4–96.4)	63.6 (57.3–69.5)	38.5 (34.2–42.9)	96.3 (92.4–98.2)
	Original set + CycleGAN	0.859 (0.803–0.914)	79.3 (67.3–88.5)	78.8 (73.2–83.6)	48.5 (41.8–55.2)	93.8 (90.2–96.1)
	Original set + StyleGAN2	0.913 (0.872–0.954)	90.4 (80.4–96.4)	75.6 (69.7–80.7)	48.3 (42.5–54.1)	96.9 (93.6–98.5)
EfficientNetB0	Original set (no augmentation)	0.796 (0.736–0.855)	71.4 (58.6–82.1)	71.6 (65.5–77.1)	38.7 (33.0–44.8)	90.8 (86.9–93.6)
	Original set + classic augmentation^a^	0.833 (0.786–0.872)	88.9 (78.4–95.4)	62.4 (56.1–68.4)	37.3 (33.2–41.6)	95.7 (91.7–97.8)
	Original set + DCGAN	0.821 (0.756–0.887)	65.0 (52.0–76.6)	88.4 (83.7–92.0)	58.5 (48.9–67.5)	90.9 (87.7–93.3)
	Original set + CycleGAN	0.875 (0.821–0.929)	71.4 (58.6–82.1)	90.4 (86.0–93.7)	65.2 (55.4–73.8)	92.6 (89.4–94.8)
	Original set + StyleGAN2	0.926 (0.890–0.963)	92.0 (82.4–97.3)	80.8 (75.3–85.4)	54.7 (48.1–61.1)	97.5 (94.5–98.9)

*CI* confidence interval, *NPV* negative predictive value, *PPV* positive predictive value, *ROC-AUC* area under the receiver operating characteristic curve

^a^We oversample the ERM class to balance the training dataset

The external validation results obtained using the RFMiD dataset are listed in Table 2. The EfficientNetB0 trained with StyleGAN2 augmentation also showed the highest AUC- 0.951 (95% CI, 0.926–0.976)-among the developed models. This model detected crystalline retinopathy with a sensitivity of 96.1% (95% CI, 80.3–99.9%), a specificity of 85.6% (95% CI, 81.6–87.2%), a PPV of 19.5% (95% CI, 16.6–22.7%), and an NPV of 99.8% (95% CI, 98.8–99.9%). Similar results were observed for other external validations using the JSIEC dataset (Table 3). EfficientNetB0 trained with StyleGAN2 augmentation also showed a detection performance with an AUC of 0.914 (95% CI, 0.818–0.999). The corresponding sensitivity, specificity, PPV, and NPV were 88.4% (95% CI, 69.8–97.5%), 94.7% (95% CI, 82.2–99.3%), 92.0% (95% CI, 74.7–97.8%), and 92.3 (95% CI, 80.5–97.2%), respectively.

**Table 2:** The prediction results from an external validation dataset (RFMiD) to detect epiretinal membrane in fundus photographs

CNN architectures	Training set	ROC-AUC (95% CI)	Sensitivity (%, 95% CI)	Specificity (%, 95% CI)	PPV (%, 95% CI)	NPV (%, 95% CI)
ResNet50	Original set (no augmentation)	0.873 (0.806–0.941)	65.3 (44.3–82.7)	90.4 (87.9–92.5)	20.9 (15.5–27.6)	98.5 (97.5–99.1)
	Original set + classic augmentation^a^	0.849 (0.820–0.875)	88.5 (69.8–97.5)	69.5 (65.8–72.9)	10.1 (8.6–11.9)	99.4 (98.1–99.8)
	Original set + DCGAN	0.914 (0.869–0.959)	88.4 (69.8–97.5)	76.8 (73.4–79.9)	12.9 (10.8–15.2)	99.4 (98.3–99.7)
	Original set + CycleGAN	0.863 (0.811–0.914)	80.7 (60.6–93.4)	79.8 (76.5–82.8)	13.4 (10.8–16.5)	99.0 (97.9–99.5)
	Original set + StyleGAN2	0.939 (0.899–0.979)	84.6 (65.1–95.6)	88.3 (85.6–90.6)	22.0 (17.8–26.8)	99.3 (98.3–99.7)
EfficientNetB0	Original set (no augmentation)	0.823 (0.759–0.886)	96.1 (80.3–99.9)	60.0 (56.2–63.8)	8.5 (7.6–9.5)	99.7 (98.3–99.9)
	Original set + classic augmentation^a^	0.829 (0.799–0.857)	80.8 (60.6–93.4)	70.4 (66.8–73.8)	9.6 (7.8–11.7)	98.9 (97.7–99.5)
	Original set + DCGAN	0.875 (0.815–0.935)	96.1 (80.3–99.9)	64.1 (60.3–67.7_	9.4 (8.4–10.5)	99.7 (98.4–99.9)
	Original set + CycleGAN	0.841 (0.773–0.908)	100 (86.7–100)	53.5 (49.6–57.3)	7.7 (7.1–8.3)	100 (98.9–100)
	Original set + StyleGAN2	0.951 (0.926–0.976)	96.1 (80.3–99.9)	84.6 (81.6–87.2)	19.5 (16.6–22.7)	99.8 (98.8–99.9)

*CI* confidence interval, *NPV* negative predictive value, *PPV* positive predictive value, *RFMiD* Retinal fundus multi-disease image dataset, *ROC-AUC* area under the receiver operating characteristic curve

^a^We oversampled the ERM class to balance the training dataset

**Table 3:** The prediction results from the external validation dataset (JSIEC) to detect epiretinal membrane in fundus photographs

CNN architectures	Training set	ROC-AUC (95% CI)	Sensitivity (%, 95% CI)	Specificity (%, 95% CI)	PPV (%, 95% CI)	NPV (%, 95% CI)
ResNet50	Original set (no augmentation)	0.808 (0.703–0.912)	88.4 (69.8–97.5)	60.5 (43.3–75.9)	60.5 (50.2–69.9)	88.4 (71.9–95.8)
	Original set + classic augmentation^a^	0.849 (0.738–0.926)	92.3 (74.9–99.1)	71.1 (54.1–84.6)	68.6 (56.7–78.4)	93.1 (77.8–98.1)
	Original set + DCGAN	0.866 (0.753–0.980)	92.3 (74.8–99.0)	76.3 (59.7–88.5)	72.7 (59.8–82.6)	93.5 (79.0–98.2)
	Original set + CycleGAN	0.879 (0.796–0.961)	69.2 (48.2–85.6)	89.4 (75.1–97.0)	81.8 (63.2–92.1)	80.9 (70.2–88.4)
	Original set + StyleGAN2	0.910 (0.817–0.983)	76.9 (56.3–91.0)	92.1 (78.6–98.3)	86.9 (68.8–95.2)	85.3 (74.1–92.2)
EfficientNetB0	Original set (no augmentation)	0.890 (0.794–0.985)	84.6 (65.1–95.6)	92.1 (78.6–98.3)	88.0 (70.9–95.6)	89.7 (77.9–95.5)
	Original set + classic augmentation^a^	0.899 (0.798–0.960)	84.6 (65.1–95.6)	94.7 (82.3–99.4)	91.7 (73.9–97.7)	90.0 (78.5–95.7)
	Original set + DCGAN	0.861 (0.764–0.959)	92.3 (74.8–99.0)	76.3 (59.7–88.5)	72.7 (59.8–82.6)	93.5 (79.0–98.2)
	Original set + CycleGAN	0.871 (0.778–0.967)	76.9 (56.3–91.0)	86.8 (71.9–95.5)	80.0 (63.2–90.2)	84.6 (72.9–91.8)
	Original set + StyleGAN2	0.914 (0.818–0.999)	88.4 (69.8–97.5)	94.7 (82.2–99.3)	92.0 (74.7–97.8)	92.3 (80.5–97.2)

*CI* confidence interval, *JSIEC* Joint Shantou International Eye Center, *NPV* negative predictive value, *PPV* positive predictive value, *ROC-AUC* area under the receiver operating characteristic curve

^a^We oversampled the ERM class to balance the training dataset

**Fig. 7:**
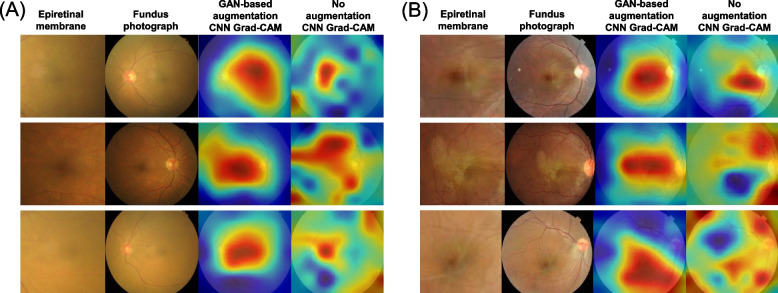
Attention maps generated by the Grad-CAM technique from the developed EfficientNetB0 to detect epiretinal membrane. **A** Healthcare center dataset. **B** External dataset (RFMiD)

To further determine whether the models properly analyzed the ERM features of the CFP, we generated attention maps of the EfficientNetB0 models using the Grad-CAM technique (Fig. 7). Using EfficientNetB0 trained with StyleGAN2 augmentation, Grad-CAM frequently focused on the central area of the macula and visualized the characteristic pathological features of ERM (cellophane reflex). EfficientNetB0, trained without GAN augmentation, frequently highlighted peripheral areas of the macula or margins of the ERM that did not match the exact location of the ERM.

Table 4 presents a comparison between the proposed method (EfficientNetB0 trained with StyleGAN2 augmentation) and recent deep learning techniques. ERM data augmentation based on the DDPM and CutMix failed to achieve a performance comparable to that of the proposed model (*P* < 0.050). The ViT model with classic data augmentation also exhibited a lower ROC AUC than the proposed model. The difference between the proposed model and the ViT trained with StyleGAN2 augmentation was not significant (*P* = 0.0914).

**Table 4:** Comparison of prediction performance from internal validation (healthcare center dataset) to detect epiretinal membrane in fundus photographs

Classification network architecture	Augmentation	ROC-AUC (95% CI)	Sensitivity (%, 95% CI)	Specificity (%, 95% CI)	*P*-value
EfficientNetB0	StyleGAN2 (Ours)	0.926 (0.890–0.963)	92.0 (82.4–97.3)	80.8 (75.3–85.4)	Reference
EfficientNetB0	DDPM [26, 27]	0.825 (0.779–0.866)	88.9 (78.4–95.4)	65.2 (58.9–71.1)	0.0048
EfficientNetB0	CutMix^a^ [28]	0.837 (0.792–0.877)	77.8 (65.5–87.3)	86.0 (81.1–90.1)	0.0080
Vision Transformer [29]	Classic augmentation^a^	0.835 (0.789–0.874)	84.1 (72.7–92.1)	75.2 (69.4–80.4)	0.0051
Vision Transformer [29]	StyleGAN2	0.863 (0.819–0.899)	82.5 (70.9–90.9)	84.0 (78.9–88.3)	0.0914

*CI* confidence interval, *DDPM* denoising diffusion probabilistic model, *ROC-AUC* area under the receiver operating characteristic curve

^a^We oversampled the ERM class to balance the training dataset

## Discussion

We aimed to synthesize CFPs with ERM using GAN techniques to address the data imbalance problem. We built an improved ERM detection model using StyleGAN2-based augmentation. Previous studies have focused on detecting ERM in CFP images using deep learning [10, 31]. However, the clinical application of the previous models was difficult because the ability to detect ERM was relatively low, and there was no external validation. Compared with previous studies, our approach additionally boosts the ERM detection performance by synthesizing CFP images using StyleGAN2, which combines normal and pathological CFPs to generate realistic synthetic images. Our study demonstrates that generative AI techniques can be used to address the lack of medical data in the CFP image domain.

Grad-CAM heatmaps showed that the proposed classification model properly analyzed the ERM features. Compared with the CNN model without augmentation, the StyleGAN2-based augmentation process focused on the location of the ERM. If a small number of training sets is used, the risk of overfitting always exists, and it is expected that the StyleGAN2 has helped to avoid overfitting. Based on this technique, our study achieved a better performance(0.926 of AUC) than that of a previous study (0.857 of AUC) in detecting ERM [10]. Several studies have developed deep learning models to detect ERM [12, 13]; however, the validation sets were different, and additional studies are needed to compare the objective performance of various deep learning models to detect ERM.

As the society ages, idiopathic ERMs are expected to occur. In addition, as the number of cataract surgeries increases, the prevalence of secondary ERM also increase [32]. Compared to the high prevalence of ERM, attempts to screen for ERMs using CFP have been relatively insufficient. Using current deep learning systems that primarily target diabetic retinopathy, age-related macular degeneration, and glaucoma [33, 34], most patients with ERM encounter diagnostic delays during the screening stage. Permanent visual damage is possible if the ERM is left unattended because there are no symptoms in the early stages. Our work establishes a deep learning model that focuses on diagnosing ERM early and shows a higher performance than traditional data learning. Table 5 presents a literature review that investigates deep learning models for ERM detection using CFP. Previous studies have reported very high performance (ROC-AUCs > 0.95) in detecting membrane features using a large dataset from a single center [11, 35]. Deep learning using large-scale multicenter datasets has also achieved high diagnostic accuracy for ERM (ROC-AUCs > 0.99) [12]. However, obtaining large-scale pathological data from ERM is difficult. Therefore, methods for achieving high accuracy with limited pathological data should be further studied. To our knowledge, no previous study has investigated a deep learning model with StyleGAN2-based augmentation for ERM detection using CFP. If our proposed generative AI method continues to expand, we can create a deep-learning model that can accurately diagnose early ERMs.

**Table 5:** A literature review for deep learning studies for detecting epiretinal membrane in fundus photography images

Study (first author, year)	Dataset	AI architecture	Summary
Son, 2020 [11]	Local clinic data (ERM, 3639 eyes) + external database	Modified ResNet + classic augmentation	The deep learning model for retinal membrane feature detection showed ROC-AUCs of 0.989 and 0.997 in two validation sets
Casado-García, 2021 [31]	A nationwide database (Spain) + RFMiD	HR-net + CycleGAN	The final model achieved a F1-score of 86.82% to detect ERM
Shao, 2021 [10]	Local clinic data (ERM, 83 eyes / no ERM, 61 eyes)	Inception-Resnet-v2 and Xception + classic augmentation	The AI model achieved an accuracy of 77.1%. It was comparable to manual reading (accuracy, 75.7%)
Kim, 2021 [36]	Local clinic data (ERM, 99 eyes / control, 79 eyes)	ResNet50 + classic augmentation	The deep learning model for ERM detection showed a sensitivity of 92.5% and specificity of 98.3%
Cen, 2021 [12]	JSIEC + LEDRS + EYEPACS	Mask R-CNN + Inception-V3, Xception, InceptionResNet-V2, and modified ResNet and ResNeXt	The final model for ERM detection showed ROC-AUCs of 0.9972 and 0.9976 in two validation sets
Li, 2022 [13]	Local clinic data (ERM, 2947 eyes)	SeResNext50 + classic augmentation	The deep learning model for ERM detection showed ROC-AUCs of 0.968 in the internal validation and 0.938 and 0.934 in the external validation
Son, 2023 [35]	Local clinic data (ERM, 3073 eyes) + MESSIDOR	EfficientNet-B7 + classic augmentation	The deep learning model for membrane feature detection showed ROC-AUCs of 0.997 in the internal validation and 0.954 in the external validation
Ours	Local clinic data (ERM, 302 eyes / control, 1250 eyes) + RFMiD + JSIEC	EfficientNetB0 + StyleGAN2	The proposed model achieved ROC-AUC of 0.926 for internal validation. ROC-AUCs of 0.951 and 0.914 were obtained for the two external validation datasets

*ERM* epiretinal membrane, *EyePACS* Eye Picture Archive Communication System Kaggle data, *JSIEC* Joint Shantou International Eye Center dataset, *LEDRS* Lifeline Express Diabetic Retinopathy Screening Systems, *RFMiD* Retinal fundus multi-disease image dataset, *ROC-AUC* area under the receiver operating characteristic curve

Currently, the CFP is the standard image domain that dominates ophthalmic screening [37]. A deep learning-based diagnosis of OCT cross-sectional images was developed for ERM. However, the detection of ERM in CFP has been overlooked. In studies using OCT, deep learning models have shown very high accuracy in detecting ERM [6, 7, 38]. The OCT, however, captures the cross-section of several local areas of the retina, so it is difficult to scan all areas of the macula with it. Therefore, early ERM may be difficult to detect with OCT. alone. In contrast, the CFP is an imaging domain that briefly depicts the entire macula. A subtle difference between the cellophane reflex of the ERM and the normal reflection of the retina exists; distinguishing between them can be difficult for ophthalmologists. Timely surgical interventions can reduce the socioeconomic costs of late-stage ERM [39]. Therefore, developing and distributing a model that accurately screens ERM through continuous development is necessary.

We addressed the challenge of using an imbalanced dataset for ERM detection. Compared with conventional linear transformation augmentation (classic augmentation), GAN-based augmentation showed improved performance in the detection of ERM. In particular, the StyleGAN2 model generated relatively high-quality and realistic CFP images. This model performed better than the CNN models using DDPM-based or CutMix augmentation methods. ViT, which recently exhibited a higher performance than CNN architectures, failed to show a better performance than the proposed CNN model with StyleGAN2 augmentation. A previous study demonstrated that StyleGAN2 can synthesize mixed-style medical images by combining the features of the training sets [17]. To learn various samples and improve the generalization of deep learning models, StyleGAN2 could be adopted for out-of-distribution sample detection of computed tomography images [40]. Our study also confirms that StyleGAN2 is a promising generative AI technique for improving medical image synthesis and prediction performance. Generative AI continues to develop by adopting and expanding various numerical and probabilistic algorithms [14]. Recent advances in diffusion methods predict the future generation of higher-quality images [36]. A recent study showed that the diffusion model outperformed GAN techniques in the CFP, chest X-ray, and histopathology imaging domains [26]. There was an attempt to improve diagnostic performance in CFP by combining GAN and Transformer structures [41], and performance improvement is expected if applied to ERM in the future.

This study has several limitations. Firstly, we generated CFP images with relatively low resolution in the GAN models, which had a resolution of 256 × 256 pixels. For the early diagnosis of ERM, it is necessary to analyze images with greater resolution. Secondly, the dataset included an East Asian population from a single healthcare center. Although the proposed model performed well on limited external validation datasets, models trained with data from a single institution are expected to degrade in performance in other clinical settings. Thirdly, the training and validation datasets included only a limited number of CFP images. Although GAN have been used to overcome the data shortage of ERM, additional data collection is essential to achieve a higher performance.

## Conclusion

We propose an improved deep learning model by synthesizing realistic CFP images with the pathological features of ERM through generative AI. We leveraged a deep learning classification model with additional StyleGAN2 training to address limited data availability. The final model outperformed the typical augmentation and other GAN-based learning methods for detecting ERM using the CFP. We believe that our deep learning framework will help achieve a more accurate detection of ERM in a limited data setting.

### Supplementary Information

**Additional file 1:** **Table S1**. Original code sources of GAN techniques. **Figure S2**. Sample anonymized color fundus photographs data with epiretinal membrane.

## Data Availability

The healthcare center data used in this study cannot be made publicly accessible owing to KNIBP restrictions. Sample-anonymized CFP data with ERM are available in the Supplementary Materials. External databases included the retinal fundus multi-disease image dataset (RFMiD, available at https://ieee-dataport.org/open-access/retinal-fundus-multi-disease-image-dataset-rfmid) [15] and the Joint Shantou International Eye Center dataset (JSIEC, available at https://www.kaggle.com/datasets/linchundan/fundusimage1000) [12].

## Abbreviations

AI: Artificial intelligence
AUC: Area under the receiver operating characteristics
CFP: Color fundus photography
CNN: Convolutional neural network
ERM: Epiretinal membrane
GAN: Generative adversarial network
JSIEC: Joint Shantou International Eye Center dataset
KNIBP: Korean National Institute for Bioethics Policy
RFMiD: Retinal fundus multi-disease image dataset
ROC: Receiver operating characteristics
OCT: Optical coherence tomography
SGD: Stochastic gradient descent
