# Supplementary material for: Development of a generative deep learning model to improve epiretinal membrane detection in fundus photography
Source: BMC Med Inform Decis Mak. 2024 Jan 26;24:25. doi: 10.1186/s12911-024-02431-4 (PMC10811871; doi:10.1186/s12911-024-02431-4)

Development of a generative deep learning model to improve epiretinal membrane detection in fundus photography

Supplementary Materials

Table S1. Original code sources of GAN techniques.

GAN architecture	Source link
DCGAN	https://github.com/dkk/DCGAN256/blob/master/Hand%20Generator.ipynb
CycleGAN	https://colab.research.google.com/github/tensorflow/docs/blob/master/site/en/tutorials/generative/cyclegan.ipynb
StyleGAN2	https://github.com/NVlabs/stylegan2-ada-pytorch<br>https://colab.research.google.com/github/parthsuresh/stylegan2-colab/blob/master/StyleGAN2_Google_Colab.ipynb

Figure S2. Sample anonymized color fundus photographs data with epiretinal membrane

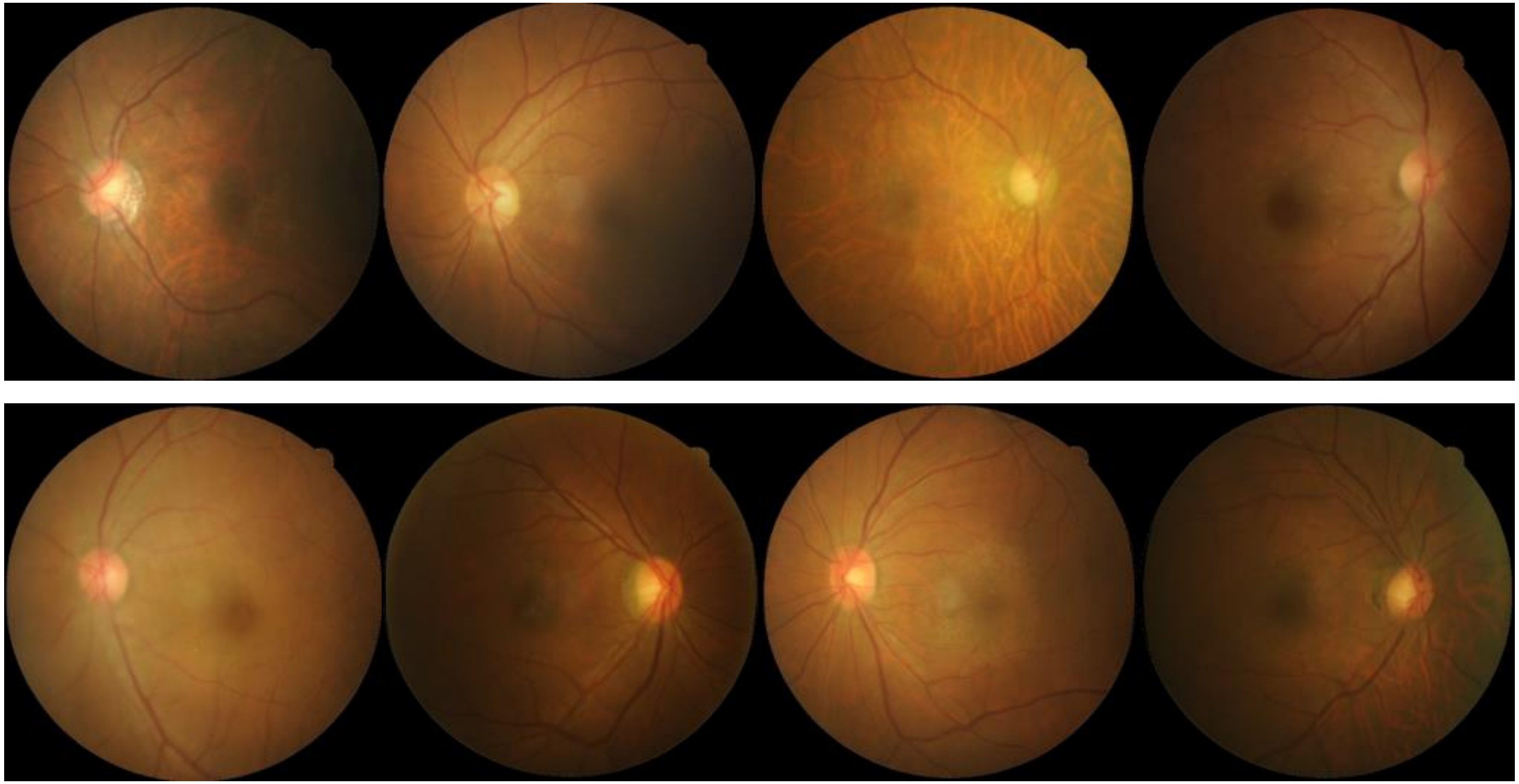

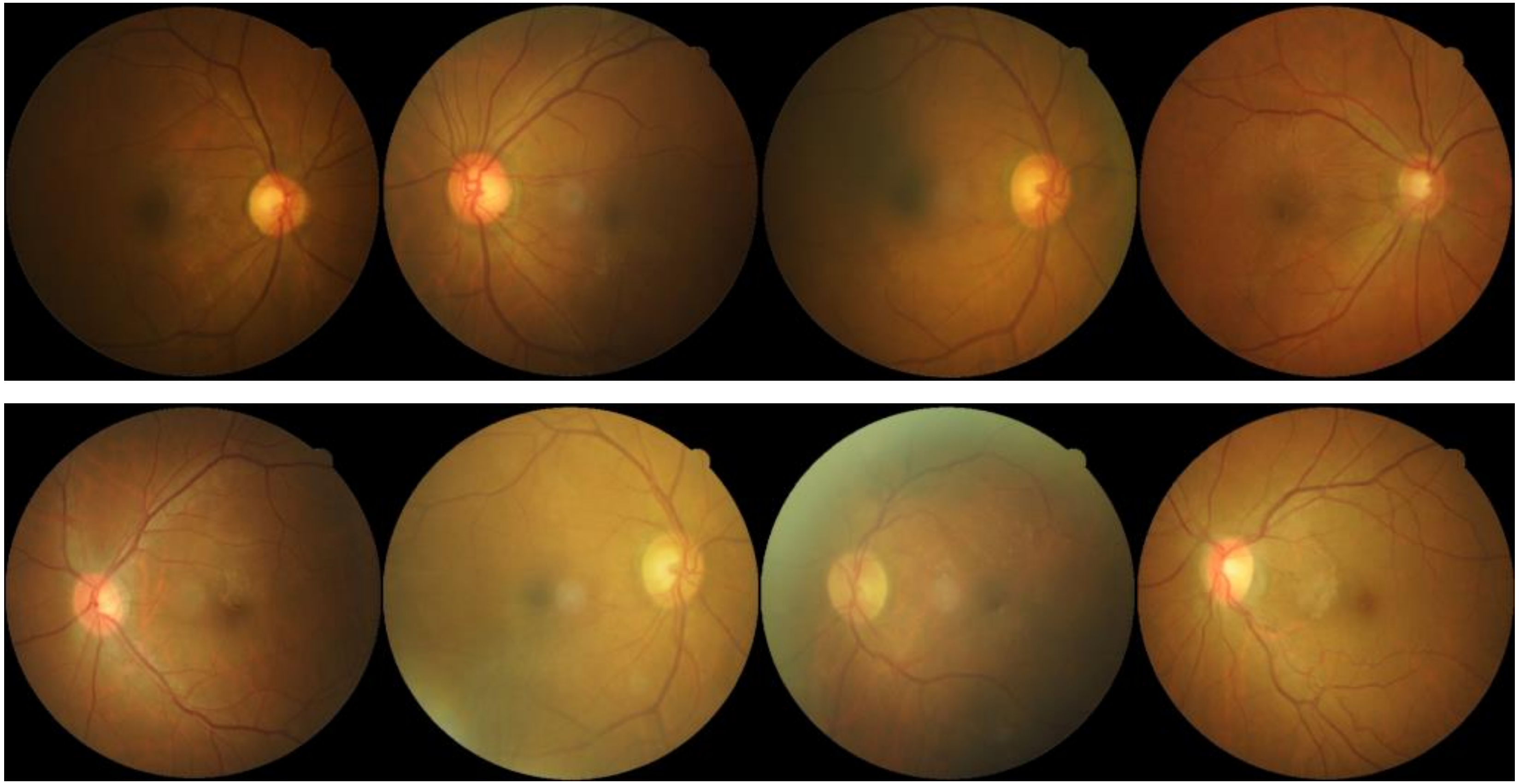

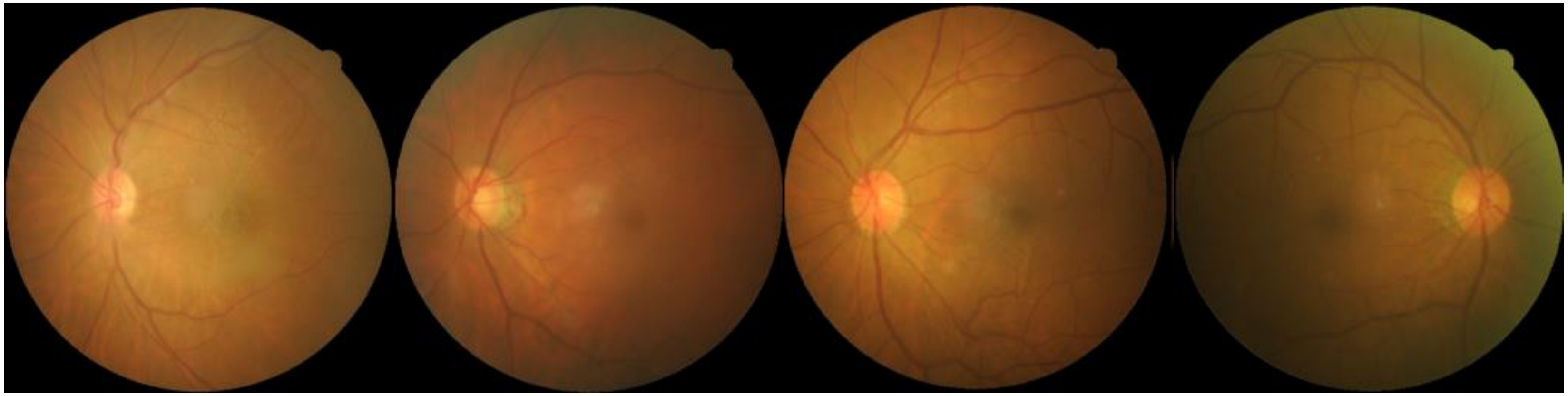

Supplement: Supplementary file 1 — Additional file 1: Table S1. Original code sources of GAN techniques. Figure S2. Sample anonymized color fundus photographs data with epiretinal membrane. [file 12911_2024_2431_MOESM1_ESM.pdf]
